# Probiotic Supplement Use among Young Children in Taiwan: A Prospective Cohort Study

**DOI:** 10.1371/journal.pone.0043885

**Published:** 2012-09-12

**Authors:** Yi-Chun Chen, Yi-Wen Chien, Pei-Jen Chang, Wu-Shiun Hsieh, Pau-Chung Chen

**Affiliations:** 1 School of Nutrition and Health Sciences, Taipei Medical University, Taipei, Taiwan; 2 Department of Nursing, National Taipei University of Nursing and Health Sciences, Taipei, Taiwan; 3 National Taiwan University Hospital and College of Medicine, National Taiwan University, Taipei, Taiwan; 4 Institute of Occupational Medicine and Industrial Hygiene, College of Public Health, National Taiwan University, Taipei, Taiwan; Tehran University of Medical Sciences, Iran (Islamic Republic of)

## Abstract

**Objectives:**

The objective of this study is to provide details on probiotic supplement use among young children in Taiwan.

**Participants and Methods:**

This study is based on the Taiwan Birth Cohort Study database. We used questionnaires to collect information on probiotic supplement use among young children from birth to 18 months of age, while also considering their demographic characteristics and other covariates. Low-birth-weight infants, preterm infants, those with birth defects, and those with caregivers who returned incomplete questionnaires were excluded. The final valid sample comprised 16,991 cases.

**Results:**

Approximately half the children received probiotic supplements before the age of 18 months. Only 6.3% of the children received probiotic supplements during the two periods of birth to 6 months and 7 to 18 months. Firstborn children, native mothers, mothers with higher educational levels, higher family income, and parents who lead healthy lifestyles were positively related to probiotic supplement use among children. Young children who were breastfed, with eczema, or with gastrointestinal tract problems were significantly positively associated with probiotic supplement use.

**Conclusion:**

The findings show that probiotic supplement usage among young children is associated with a more socially advantaged circumstance and certain child health factors, such as eczema, diarrhea, and constipation. Parents might use probiotic supplements for prevention or treatment of child diseases. The findings of this research could serve as a baseline for future studies, and provide insight into probiotic supplement use behavior for health professionals caring for infants and young children.

## Introduction

Probiotics are defined as live microbial food supplements that benefit the host by improving the gut flora [1]. Probiotics are currently used in numerous countries as food additives and supplements. These products are increasing in popularity and usage throughout the developed world, such as in Japan, Europe, and the United States [2]–[4]. Probiotics are culturally acceptable for use in Taiwan. Yakult, a Japanese probiotic milk product, is extremely popular among young children in Taiwan. Over the past few years, the number of studies supporting the health benefits of feeding probiotics to infants and children has increased. The benefits of probiotics include maintaining the intestines in good health, improving lactose intolerance [5], decreasing the frequency of infant diarrhea [6], and preventing and managing allergies [7]. In addition to probiotic supplements, several countries have marketed formula and other foods supplemented with probiotics [8]–[10].

In most countries, probiotic supplements are typically regulated as dietary supplements rather than as pharmaceutical or biological products [11]. Thus, demonstrating the safety, purity, or potency is usually not required before marketing probiotics. This can lead to significant inconsistencies between the stated and actual content of probiotic products [12]. In Europe, dietary supplements intended for infants and children have specific compositional legal requirements [13]. In Japan, probiotic products marketed for specified health usage require a formal premarket review by the Minister of Health and Welfare [14]. In the United States, dietary supplements do not require premarket approval from the Food and Drug Administration [15]. In Taiwan, probiotic products marketed for specific health benefits require a premarket review by the Food and Drug Administration. Although most commercially available probiotic strains are widely considered safe, concerns about their consumption among particular populations, such as infants and children, remain.

Accumulating evidence of the benefits probiotics provide has led to greater consumption of probiotic supplements. However, data on probiotic supplementation in the pediatric population are scant. The objective of this study was to provide details on probiotic supplement usage among children in Taiwan and to determine the predictors of probiotic supplement consumption in this population.

## Materials and Methods

### Study Design

The aim of the Taiwan Birth Cohort Study (TBCS) is the development of a nationally representative, cohort database to establish national norms of psychosocial measurement. Prior to the formal TBCS, a pilot study (TBCS-p) with a random sampled cohort of 2048 was conducted. The pilot study involved evaluating the sampling design and procedure, developing and testing the adequacy of research instruments, identifying potential fieldwork problems, and assessing the overall study protocol [16]. The Taiwan Birth Cohort Study is a prospective longitudinal cohort study that involves using a stratified multi-stage systematic sampling design to obtain representative samples from the 2005 Taiwan national birth registration data. A total of 369 towns were sorted into 12 strata, based on administrative divisions (four strata) and fertility rates (three strata). Using the principle of probability proportionate to size, we randomly selected 85 primary sampling units (from 90 of 369 towns) from the 12 strata. A total of 24 200 pairs of parents and newborns were recruited to participate in the study. The study was approved by the Ethics Review Board of the College of Public Health, National Taiwan University. Before the home interview, the researchers in this study delivered cards to notify the participating women about the interview and invited them to participate in the survey. After the women agreed to participate, the interviewers visited the women and their families at their homes, explained the details of the study, and asked the mothers to provide written informed consent. The participants were free to withdraw at any time without having to provide a reason. Strict confidentiality was maintained throughout the process of data collection, entry, and analysis.

### Study population

Basic demographic information of the parents and infants was obtained from Taiwan's 2005 national birth registry. We conducted home interviews with postpartum parents at 6 months and at 18 months, using a structured questionnaire. Data about child growth, development and health status, child care and lifestyle, and family environment were collected by the questionnaire. Each part of the questionnaire was reviewed and revised by several health professionals. The preliminary questionnaire was further revised after it was used in the pilot study (TBCS-p). There were a total of 4028 cases (2952 from the first interview and 1076 from the second interview) lost to follow-up because of refusal to participate, home relocation, incorrect address, infant death, and other reasons. A total of 20172 (83.4%) women completed the survey, including two rounds of interviews. A total of 2396 cases involving infants with birth defects, birth weight less than 2500 g, preterm infants (less than 37 gestational weeks at birth), and those with birth defects were excluded from the study; 785 incomplete questionnaires were also excluded from the study. Consequently, the following data analysis was based on 16991 cases.

### Probiotic Supplement Usage

Probiotic-related data were obtained from the child care and lifestyle part of the interview questionnaire. Probiotic supplement users were defined according to responses to the questions “Have you given probiotic supplements, such as lactic acid-producing bacteria or *Bifidobacterium*, to your child since their birth?” (at the 6-month interview) and “Have you ever given probiotic supplements, such as lactic acid-producing bacteria or *Bifidobacterium*, to your child in the past year?” (at the 18-month interview). Dairy probiotic products, such as yogurt and probiotics enriched formula milk, were not considered to be probiotic supplements in this study. Cases were categorized into 4 groups: nonusers (never used probiotic supplements), 0 to 6 months users (receiving probiotic supplements between 0 and 6 months of age, but not receiving between 7 and 18 months of age), 7 to 18 months users (not receiving probiotic supplements between 0 and 6 months of age, but receiving between 7 and 18 months of age), and 0 to 18 months users (receiving probiotic supplements for 2 periods; 0 to 6 months and 7 to 18 months).

### Independent Variables

Data on potential predictors, including children's demographic characteristics, dietary factors, health status factors, and lifestyle factors, were obtained from Taiwan's national birth registry and interview questionnaires. Probiotic supplements can be defined as one type of dietary supplement. We chose these potential predictors according to previous studies related to children dietary supplementation [17]–[21].

Children were grouped into 2 categories according to the following parity: firstborn and non-firstborn. The mothers' nationalities were classified as either Taiwanese or foreign. The mothers' educational levels were categorized into 2 groups: university/college or above and high school or below; monthly family income was categorized into 4 groups in New Taiwan dollars (NT$), less than NT$50000; from NT$50000 to NT$70000; from NT$70000 to NT$100000; and NT$100000 and above (US$1 = approximately NT$32 in 2005); and household urbanicity was categorized into 3 groups: urban, main street in a rural area, and rural.

Lifestyle factors included the children's television watching time and parents' lifestyles. The children's television watching time was classified into 2 categories: less than 2 h per day and more than 2 h per day. The parents' lifestyles were classified into 2 categories: a healthy lifestyle (both parents had no smoking, drinking, or betel nut-chewing habits) and an unhealthy lifestyle (either parent had a smoking, drinking, or betel nut-chewing habit).

The children's dietary factors included infant feeding patterns and follow-up formula intake at 18 months. Infant feeding types were classified into 3 groups: formula-fed (never breastfed), breastfed less than 6 months, and breastfed more than 6 months. Follow-up formula intake was classified into 2 groups: high intake of follow-up formula (more than 5 times per week) and low intake of follow-up formula (equal to or less than 5 times per week).

Health-related factors included family allergy history, children's eczema, frequency of constipation as determined by diagnosis, and frequency of diarrhea as determined by diagnosis. Family allergy history (defined as the parent having either asthma, atopic dermatitis, or allergic rhinitis) were classified into groups of “yes” and “no.” Children's eczema statuses (defined as the child having either atopic dermatitis or seborrhea dermatitis) were classified into 4 groups: no eczema, had eczema from 0 to 6 months of age, had eczema from 7 to 18 months of age, and continually had eczema from 0 to 18 months of age. Information on eczema status was based on physician diagnosis provided within 6 and 18 months according to the parent reports. Constipation frequency, as determined by physician diagnoses, was grouped into 3 categories: never, once or twice per year, and more than 3 times per year. Diarrhea frequency, as determined by physician diagnoses, was grouped into 3 categories: never, once or twice per year, and more than 3 times per year. Information on constipation and diarrhea was based on physician diagnosis from 7 to 18 months of age and reported by the parents at the 18-month mark of the interviews.

### Analysis

A chi-square test was performed to assess the differences in demographic characteristics, diet-related factors, health statuses, and lifestyles potentially related to probiotic usage among children of various groups (nonuser, using between 0 and 6 months only, using between 7 and 18 months only, and using between both 0 and 6 months and 7 and 18 months). Multinomial logistic regression and logistic regression were performed to estimate the odds ratio (OR) of probiotic supplement usage with a 95% confidence interval (CI), following adjustment for potential predictors. The statistical threshold for significance was set at *P* = .05. Statistical analysis was conducted using the SPSS (Version 15.0; SPSS Inc., Chicago, IL, USA).

## Results

### Prevalence of probiotic supplements

Table 1 shows the prevalence of probiotic supplements according to different characteristic variables. Approximately half the children had received probiotic supplements. Roughly 10% of these children received probiotic supplements from birth to 6 months of age and had not received probiotic supplements after 6 months of age. However, more than one third of the children had received probiotic supplements between 7 and 18 months of age. Only 6.3% of the children had received probiotic supplements from birth to 6 months of age and between 7 and 18 months of age. Approximately 40% of the children had received probiotic supplements after 6 months of age.

**Table 1:** Probiotic supplement use according to different characteristic variables.

	nonuser	0–6 mo user	7–18 mo user	0–18 mo user	*P*
**Prevalence**	8,582(50.5)	1,616(9.5)	5,720(33.7)	1,073(6.3)	
**Demographic characteristics**					
**Birth order**					<0.001
**Non first-born**	4,347 (52.8)	811 (9.9)	2,592 (31.5)	477 (5.8)	
**First-born**	4,235 (48.3)	805 (9.2)	3128 (35.7)	596 (6.8)	
**Maternal education**					<0.001
**≦High school**	5,024 (54.8)	927 (10.1)	2,697 (29.4)	524 (5.7)	
**≧University**	3,558 (45.5)	689 (8.8)	3,023 (38.7)	549 (7.0)	
**Maternal country**					<0.001
**Foreign**	1,503 (67.5)	282 (12.7)	366 (16.4)	77 (3.5)	
**Taiwanese**	7,079 (48.0)	1,334 (9.0)	5,354 (36.3)	996 (6.7)	
**Family income**					<0.001
**<50,000**	3,875 (55.3)	767 (10.9)	1,971 (28.1)	398 (5.7)	
**50,000∼70,000**	2,147 (48.2)	399 (9.0)	1,614 (36.2)	294 (6.6)	
**70,000∼100,000**	1,676 (45.9)	297 (8.1)	1,408 (38.6)	269 (7.4)	
**>100,000**	884 (47.1)	153 (8.2)	727 (38.8)	112 (6.0)	
**Urbanicity**					<0.001
**Rural**	2,262 (53.4)	383 (9.1)	1,343 (31.7)	244 (5.8)	
**Main street in a rural area**	2,437 (51.4)	520 (11.0)	1,462 (30.8)	324 (6.8)	
**Urban**	3,883 (48.4)	713 (8.9)	2,915 (36.4)	505 (6.3)	
**Lifestyle factor**					
**Children TV time**					0.011
**<2 hr/d**	5,199 (51.4)	972 (9.6)	3,309 (32.7)	631 (6.2)	
**≧2 hr/d**	3,383 (49.2)	644 (9.4)	2,411 (35.0)	442 (6.4)	
**Parent's lifestyle**					<0.001
**Unhealthy**	5,857 (52.1)	1,081 (9.6)	3,620 (32.2)	691 (6.1)	
**Healthy**	2,725 (47.5)	535 (9.3)	2,100 (36.6)	382 (6.7)	
**Dietary factor**					
**Infant feeding pattern**					<0.001
**Formula fed**	1,528 (55.4)	300 (10.9)	793 (28.8)	136 (4.9)	
**Breastfed<6 mo**	4,950 (48.6)	938 (9.2)	3,615 (35.5)	685 (6.7)	
**Breastfed≧6 mo**	2,104 (52.0)	378 (9.3)	1,312 (32.4)	252 (6.2)	
**Follow-up formula intake**					0.017
**High intake**	7,717 (50.9)	1,449 (9.6)	5,049 (33.3)	954 (6.3)	
**Low intake**	865 (47.5)	167 (9.2)	671 (36.8)	119 (6.5)	
**Health factor**					
**Family allergy history**					<0.001
**No**	5,856 (50.6)	1,033 (8.9)	3,997 (34.5)	693 (6.0)	
**Yes**	2,726 (50.4)	583 (10.8)	1,723 (31.8)	380 (7.0)	
**Children eczema status**					<0.001
**No**	6,234 (51.4)	1,129 (9.3)	4,007 (33.1)	752 (6.2)	
**0–6 months**	1,682 (50.1)	377 (11.2)	1,099 (32.7)	201 (6.0)	
**7–18 months**	496 (42.8)	90 (7.8)	481 (41.5)	91 (7.9)	
**0–18 months**	170 (48.3)	20 (5.7)	133 (37.8)	29 (8.2)	
**Frequency of constipation**					<0.001
**Never**	7,481 (51.6)	1,395 (9.6)	4,739 (32.7)	871 (6.0)	
**≦2 times/yr**	845 (45.7)	171 (9.2)	692 (37.4)	142 (7.7)	
**≧3 times/yr**	256 (39.1)	50 (7.6)	289 (44.1)	60 (9.2)	
**Frequency of diarrhea**					<0.001
**Never**	4,213 (52.7)	821 (10.3)	2,495 (31.2)	465 (5.8)	
**≦2 times/yr**	3,296 (49.8)	605 (9.1)	2,282 (34.5)	436 (6.6)	
**≧3 times/yr**	1,073 (45.1)	190 (8.0)	943 (39.7)	172 (7.2)	

*P*<0.05 is significantly different.

Several demographics, including lifestyle, children's diet, and health variables, were associated with probiotic supplement usage. The prevalence of nonusers and supplement usage between 0 and 6 months of age was extremely similar among most characteristic groups. The prevalence of supplement usage between 7 and 18 months of age and between 0 and 18 months of age was extremely similar in most characteristic groups. Firstborn children were more likely to have received probiotics between 7 and 18 months of age and between 0 to 18 months of age. Foreign mothers, mothers with the highest education levels at high school or below were less likely to provide probiotic supplements to their children from birth or after the age of 6 months. Children from families with a higher household income and living in an urban area were more likely to receive probiotic supplements between 7 and 18 months of age and between 0 and 18 months of age. Children who spent more time watching television and had parents who demonstrated a healthy lifestyle had significantly higher probiotic supplement usage after 6 months of age. Probiotic supplement usage was lowest in the formula-fed group and high follow-up formula intake group. Children with a family history of eczema were more likely to receive probiotic supplements from birth. Children's health status was significantly related to their probiotic supplement usage. Children without eczema were less likely to receive probiotic supplements. Children with eczema between 0 and 18 months of age were more likely to receive probiotic supplements at this age. Children who experienced constipation and diarrhea more than 2 times per year after 6 months of age had significantly higher probiotic supplement usage rates after 6 months of age.

### Predictors of probiotic supplement use

Tables 2 shows the multivariate model analyses of adjusted ORs with 95% CIs on probiotic supplement usage. After adjusting for other potential confounding variables, children having lived on main streets in rural areas and with eczema between 0 and 6 months of age were 1.2 times more likely to receive probiotic supplements between 0 and 6 months of age (*P*<.05). After adjusting for other potential confounding variables, firstborn children, mothers with higher educational levels, and Taiwanese mothers were significantly positively related to probiotic supplement usage between 7 and 18 months and between 0 and 18 months of age. Infants who were breastfed, with eczema between 7 and 18 months of age, and had a higher frequency of constipation and of diarrhea were significantly positive related to probiotic supplement usage after 6 months of age (*P*<.05). Children's television watching time, parents' healthy lifestyles, and follow-up formula intake were only significantly positively related to probiotic supplement usage between 7 and 18 months of age (*P*<.05). Family income and family allergy history have different effects on probiotic supplement usage among different age ranges. Compared to those with a family income of less than NT$50 000, children with a higher family income were approximately 1.2 times more likely to receive probiotic supplements between 7 and 18months and between 0 and 18 months of age, but 20% less likely to receive probiotic supplements only between 0 and 6 months of age. Compared to children without a family history of allergies, children with such a history were 1.2 times more likely to receive probiotic supplements from birth, but 10% less likely to receive probiotic supplements only between 7 and 18 months of age.

**Table 2:** Adjusted odds ratios and confidence intervals of probiotic supplement use.

	0–6 mo user/Nonuser	7–18 mo user/Nonuser	0–18 mo user/Nonuser
	OR(95% CI)	OR(95% CI)	OR(95% CI)
**Demographic characteristic**			
**Birth order**			
**Non first-born** †	1	1	1
**First-born**	1.00(0.90–1.11)	1.23(1.15–1.32)***	1.26(1.11–1.44)***
**Maternal education**			
**≦High school** †	1	1	1
**≧University**	1.13(0.99–1.28)	1.19(1.10–1.29)***	1.17(1.00–1.36)*
**Maternal country**			
**Foreign** †	1	1	1
**Taiwanese**	1.03(0.88–1.20)	2.58(2.27–2.94)***	2.46(1.91–3.16)***
**Family income**			
**<50,000** †	1	1	1
**50,000∼70,000**	0.90(0.79–1.04)	1.22(1.11–1.33)***	1.11(0.94–1.31)
**70,000∼100,000**	0.83(0.71–0.98)*	1.22(1.10–1.34)***	1.17(0.98–1.41)
**>100,000**	0.81(0.66–0.99)*	1.18(1.04–1.34)**	0.92(0.73–1.18)
**Urbanicity**			
**rural** †	1	1	1
**main street in a rural area**	1.26(1.09–1.45)**	1.00(0.91–1.10)	1.23(1.03–1.46)*
**urban**	1.08(0.94–1.23)	1.18(1.08–1.29)***	1.13(0.96–1.34)
**Lifestyle factor**			
**Children TV time**			
**<2 hr/d** †	1	1	1
**≧2 hr/d**	1.01(0.91–1.13)	1.12(1.05–1.21)**	1.07(0.94–1.22)
**Parent's lifestyle**			
**Unhealthy** †	1	1	1
**Healthy**	1.07(0.95–1.20)	1.09(1.01–1.17)*	1.06(0.92–1.22)
**Dietary factor**			
**Infant feeding pattern**			
**Formula fed** †	1	1	1
**Breastfed<6 mo**	0.96(0.83–1.11)	1.26(1.14–1.39)***	1.44(1.18–1.75)***
**Breastfed≧6 mo**	0.89(0.75–1.05)	1.12(1.00–1.26)	1.31(1.04–1.64)*
**Follow-up formula intake**			
**High intake** †	1	1	1
**Low intake**	1.05(0.88–1.25)	1.14(1.02–1.28)*	1.08(0.88–1.33)
**Health factor**			
**Family allergy history**			
**No** †	1	1	1
**Yes**	1.20(1.07–1.34)**	0.91(0.84–0.98)*	1.17(1.02–1.34)*
**Children eczema status**			
**No** †	1	1	1
**0–6 months**	1.21(1.06–1.38)**	1.01(0.92–1.10)	0.96(0.81–1.13)
**7–18 months**	1.01(0.80–1.27)	1.31(1.15–1.50)***	1.34(1.06–1.70)*
**0–18 months**	0.64(0.40–1.02)	1.00(0.79–1.27)	1.17(0.78–1.75)
**Frequency of constipation**			
**Never** †	1	1	1
**≦2 times/yr**	1.08(0.91–1.29)	1.34(1.20–1.49)***	1.48(1.22–1.79)***
**≧3 times/yr**	1.04(0.77–1.42)	1.84(1.54–2.20)***	2.05(1.53–2.75)***
**Frequency of diarrhea**			
**Never** †	1	1	1
**≦2 times/yr**	0.94(0.84–1.06)	1.18(1.09–1.27)***	1.20(1.04–1.38)*
**≧3 times/yr**	0.91(0.77–1.09)	1.53(1.38–1.70)***	1.48(1.22–1.78)***

† Reference group.

* *P*<0.05.

** *P*<0.01.

*** *P*<0.001.

Table 3 shows predictors of probiotic supplement usage between 7 and 18 months of age for infants who had ever received probiotic supplements between 0 and 6 months of age. Family income, infant feeding patterns, and children's health status were significantly related to probiotic supplement usage between 7 and 18 months of age (*P*<.05). Compared to formula-fed infants, those who were breastfed were 1.5 times more likely to have received probiotic supplements again between 7 and 18 months of age. Compared to infants without eczema, infants with allergies only between 0 and 6 months of age were 23% less likely to have received probiotic supplements again between 7 and 18 months of age, but infants with allergies at 0 to 18 months of age were 1.9 times more likely to have received probiotic supplements again between 7 and 18 months of age. Compared to children without gastrointestinal tract problems between 7 and 18 months of age, children who experienced a higher frequency of constipation or diarrhea were 1.3–2.0 times more likely to have received probiotic supplements again between 7 and 18 months of age.

**Table 3:** Predictors of probiotic supplement usage between 7 and 18 months.

	0–18 mo user/0–6 mo user	
	OR	(95% CI)
**Demographic characteristic**		
**Birth order**		
**Non first-born** †	1	
**First-born**	1.25	(1.06–1.46)*
**Maternal education**		
**≦High school** †	1	
**≧University**	1.03	(0.86–1.25)
**Maternal country**		
**Foreign** †	1	
**Taiwanese**	2.43	(1.83–3.24)***
**Family income**		
**<50,000** †	1	
**50,000∼70,000**	1.20	(0.98–1.48)
**70,000∼100,000**	1.41	(1.12–1.77)**
**>100,000**	1.11	(0.82–1.50)
**Urbanicity**		
**rural** †	1	
**main street in a rural area**	0.95	(0.76–1.19)
**urban**	1.07	(0.87–1.32)
**Lifestyle factor**		
**Children TV time**		
**<2 hr/d** †	1	
**≧2 hr/d**	1.04	(0.88–1.22)
**Parent's lifestyle**		
**Unhealthy** †	1	
**Healthy**	0.98	(0.83–1.17)
**Dietary factor**		
**Infant feeding pattern**		
**Formula fed** †	1	
**Breastfed<6 mo**	1.49	(1.18–1.88)**
**Breastfed≧6 mo**	1.45	(1.10–1.90)**
**Follow-up formula intake**		
**High intake** †	1	
**Low intake**	1.07	(0.83–1.39)
**Health factor**		
**Family allergy history**		
**No** †	1	
**Yes**	0.96	(0.81–1.14)
**Children eczema status**		
**No** †	1	
**0–6 months**	0.77	(0.63–0.95)*
**7–18 months**	1.28	(0.93–1.76)
**0–18 months**	1.88	(1.04–3.40)*
**Frequency of constipation**		
**Never** †	1	
**≦2 times/yr**	1.36	(1.06–1.74)*
**≧3 times/yr**	2.02	(1.36–3.02)**
**Frequency of diarrhea**		
**Never** †	1	
**≦2 times/yr**	1.29	(1.09–1.54)**
**≧3 times/yr**	1.60	(1.26–2.04)***

† Reference group.

* *P*<0.05.

** *P*<0.01.

*** *P*<0.001.

## Discussion

Numerous studies have described the health effects that probiotics have on children [8], [10], [22], [23], but few have analyzed the factors that influence their use. The results of this study revealed that approximately half of the TBCS population had used probiotic supplements before 18 months of age. Approximately 40% of this population had used probiotic supplements between 7 and 18 months of age, and it was higher than 15.6% at 6 months of age in another study [24]. The difference may be partly due to the longer study period (1 year) for TBCS data at 18 months, as compared with the shorter study period (6 months) for TBCS data at 6 months. It is possible that the first questionnaire at 6 months prompted the parents to begin administering probiotic supplements. Additionally, the parents in this study tended to feed their children more probiotics as they matured. The findings showed that only 6.3% of the TBCS population used probiotics from 0 to 18 months of age, and approximately 10% of infants who had consumed probiotic supplements when aged between 0 and 6 months discontinued consumption as they matured.

The findings revealed that probiotic supplement usage among young children is associated with a more socially advantaged circumstance and healthier lifestyle, such as those of firstborn children, native mothers, mothers with higher educational levels, mothers with a higher family income, and parents who lead healthy lifestyles. The results are consistent with those of previous studies examining other nutrient supplements. Two previous studies have similarly shown that firstborn children were fed more dietary supplements than non-firstborn children [17], [20]. Parents have more time and money to spend on their firstborn child. As the number of children in a family increases, disposable income for non-essential products such as probiotic supplements decreases. Furthermore, if parents believe that probiotics do not influence the health of their firstborn, they may discontinue probiotic use for subsequent children. The consumption rate for probiotic supplements was higher for children with Taiwanese mothers and for those who live in urban areas. Vitamin and mineral supplement surveys have also reported similar demographic associations [17], [18], [21]. The findings of this study showed that parents leading healthy lifestyles are more likely to provide probiotic supplements to their children than parents with unhealthy lifestyles. This finding is in accordance with the results of dietary supplementation and healthier lifestyle behaviors among adults [19], [25].

Watching television for more than 2 h per day might be considered an unhealthy activity for children. For children's health, the American Academy of Pediatrics suggests that parents discourage television viewing for children less than 2 years of age, and that parents limit older children's total media time to 1–2 h per day [26]. Previous studies have shown that vitamin and mineral supplement usage among children is positively associated with less television viewing time among children [21], [27]. The findings of this study are not consistent with those of previous studies. The usage rate of probiotic supplements between 7 and 18 months of age was higher among children who had watched television for more than 2 h per day, as compared to children who had watched television for less than 2 h per day. Children's television viewing time was positively related to parents' television watching time [28]. In Taiwan, television shopping channels have become popular in recent years. Parents whose children watch television for more than 2 h per day may also be exposed to more probiotic supplement advertisements, which may reinforce the belief that they should provide their children with probiotic supplements. Further research is required to explore associations between the use of probiotic supplements and media exposure.

The results indicate that breastfed children are more likely to receive probiotic supplements than formula-fed children, especially after 6 months of age. The usage rate of probiotic supplements is also higher among children with a lower intake of follow-up formula than among children with a higher intake of follow-up formula. Certain substances, such as bifidus factor in breast milk, can stimulate the growth of beneficial bacteria [29]. Previous studies have noted that breast milk may be a source of beneficial bacteria that colonize infants' gastrointestinal tracts [30], [31]. However, an increasing number of follow-up formulas and toddler formulas supplemented with probiotics have been promoted in Taiwan, as well. For young children who were breastfed or fed with a low intake of follow-up formula, their parents may be concerned about the possibility of insufficient intake of probiotics by their children, leading the parents to provide probiotic supplements.

We found that parents with a history of allergies were more likely to feed probiotic supplements to their children from birth; such a finding is consistent with the results of a previous study on probiotic usage among children aged between 0 and 6 months [24]. These findings suggest that parents with allergies may provide their children with probiotic supplements for prevention of allergies. Children's eczema status and frequency of constipation and diarrhea were positively related to probiotic usage. The proposed health benefits of probiotics have been subjected to increasingly rigorous scientific evaluation in recent years. Two meta-analyses confirmed that probiotic use reduces risk of atopic dermatitis among children in the first 2 years after birth [32], [33]. Kalliomaki, Antoine, and Herz et al. indicated that, at present, a specific probiotic strain cannot be recommended for the general treatment of eczema or atopic eczema [7]. A recent Cochrane review suggested that probiotics are safe and provide clear benefits by reducing the duration and stool frequency of acute infectious diarrhea [34]. After a systematic review of random controlled trials, Chmielewska and Szajewska determined that data published thus far provide insufficient scientific evidence to support a general recommendation for the use of probiotics to treat functional constipation [35]. Emphasizing that probiotics are highly heterogeneous and vary in composition, biological activity, dosage, and preparation is critical [36]. Furthermore, crucial questions remain before establishing a clinical application of probiotics, including the optimal duration of probiotic administration and the preferred microbial dose and species [22], [23].

The findings showed that an association exists between probiotic supplement usage and certain child health factors, such as eczema, diarrhea, and constipation. In Taiwan, people can easily purchase probiotic supplements in certain clinics and drugstores. Parents might use probiotic supplements for prevention or treatment of child diseases. Although probiotics have an excellent overall safety record, safety concerns exist for the use of probiotics in infants and children who are immunocompromised, chronically debilitated, or seriously ill with indwelling medical devices [12], [23]. Boyle, Robin-Brown, and Tang reviewed several studies and noted that numerous case reports describe clinical sepsis related to probiotics use [12]. A greater understanding of the short- and long-term consequences of introducing a foreign bacterial strain (probiotics) remains a significant challenge for the future of pediatric nutrition research [23].

One potential limitation of this study is incomplete or inaccurate reporting. Radimer noted that longer time frames may be more effective for capturing usual dietary supplement intake, but imprecise memory may diminish this advantage [37]. This study relied on single parents to report the probiotics consumed by children, and may have overlooked exposures that were unknown to the parents. Because long-term (6-month and 1-year) use was surveyed, possible recall errors could have occurred. To reduce recall bias, we recruited only participants who had completed an interview at 6 and 18 months, and we examined whether they used probiotic supplements, without including frequency or dose. The other limitation was that information on motivation of probiotic supplement usage was not collected.

This study provides recent population-based data on probiotic supplement usage among children in Taiwan. The results revealed that a higher socioeconomic status, breastfeeding, lower intake of follow-up formula, and eczema are positively related to using probiotic supplements. The findings of this study could serve as a baseline for future studies, and provide insight into probiotic supplement usage for health professionals caring for infants and young children.
